# Disclosure experience to partner and its effect on intention to utilize prevention of mother to child transmission service among HIV positive pregnant women attending antenatal care in Addis Ababa, Ethiopia

**DOI:** 10.1186/1471-2458-13-765

**Published:** 2013-08-17

**Authors:** Endalew Gemechu Sendo, Amsale Cherie, Tadese Asfaw Erku

**Affiliations:** 1 Addis Ababa University, College of Health Science, School of Nursing and Midwifery, Addis Ababa, Ethiopia, P.O. Box 1176, Ethiopia; 2 Nursing Department, Debre Markos University, Health Science College, Debre Markos, P.O.Box 269, Ethiopia

**Keywords:** HIV status disclosure, PMTCT, Sexual partner

## Abstract

**Background:**

Disclosure of HIV status has become an entry criterion for prevention of mother to child transmission programs in resource-constrained countries. However, little has been explored about the prevalence of, barriers to, outcomes and factors associated with HIV status disclosure among HIV positive pregnant women in Ethiopia.

**Method:**

Cross- sectional study was conducted among 107 pregnant women who were attending antenatal care in public centers from April to June 2011 in Addis Ababa capital city of Ethiopia.

Data was collected using interviewer administered pretested structured questionnaire and entered and was analyzed using SPSS- 15 version. Results presented in tables.

**Result:**

Seventy three percent of women had disclosed their HIV status to their partner. Discussion about testing and a smooth relationship with the partner were independently associated with their disclosure. Women who disclosed to their partners were almost five times more likely to participate in Prevention of Mother to Child Transmission programs than their counterparts (AOR = 4.74; 95% CI 1.24-18.14).

**Conclusions:**

Although most participants disclosed their HIV sero-positive status, lack of disclosure by some women might result in a limited ability to participate in PMTCT programs. Thus, assertiveness and improved communication skills training should be offered to HIV positive pregnant mothers and be reinforced during on-going counseling.

## Background

Disclosure of HIV status has become an entry criterion for prevention of mother to child transmission programs in resource-constrained countries [1,2]. However, little has been explored about the prevalence of, barriers to, outcomes and factors associated with HIV status disclosure among HIV positive pregnant women in Ethiopia.

Disclosure benefits both the individual and the public. To the individual, benefits include less anxiety and [1,3] increased social support. Studies showed that there are (l) about 2.3 million children under 15 years living with AIDS in the world, and the vast majority of these were in Sub-Saharan Africa.

Mother-to-child transmission of HIV is associated with up to 90% of all infection in children up to six years [4,5]. It is estimated that without any intervention about 35% of children born to HIV infected mothers will be infected with the virus. This percentage has reportedly been reduced to levels as low as 2% in developed countries with the advent of antiretroviral drugs and the implementation of core PMTCT intervention [6,7].

In addition, a larger proportion of studies from developing countries reported that women did not share their HIV test results with anyone (10%-78%) as compared to women in developed countries (3%-10%) [8-13]. The prevalence of HIV positive pregnant women in Addis Ababa is 7.2% [9]. There are minimal studies regarding disclosure of HIV status in the study area. Therefore, this present study is undertaken to determine the prevalence of, barriers to, and effects of HIV positive status disclosure among HIV positive pregnant women attending ANC in Public health Health centers in Addis Ababa, Ethiopia”.

## Methods

### Study design and area

An institution- based cross-sectional study was conducted from April to June 2011 among HIV positive pregnant women attending ANC public centers in Addis Ababa capital city of Ethiopia.

### Sample size and sampling technique

A single population proportion formula, [n = (Z α/2)2 p (1-p) / d2], was used to estimate the sample size. The following assumptions were made: prevalence of HIV positive pregnant women in Addis Ababa 7.2%, [3] 95% confidence interval, margin of error 5% (d = 0.05) and allowance for non-response 10%. Then the total sample size calculated was 112. All eight health centers that have a client flow of 4–10 clients per month in the city were included in the study. From the selected health centers, all HIV positive pregnant women who gave their consent to participate were interviewed.

### Data collection

Structured interviewer questionnaire was developed to collect information about the dependent variable regarding HIV positive status disclosure to a partner and intension of utilization of PMTCT and characteristics that determine disclosure of HIV status namely socio-demographic characteristics (age, income, education, religion and occupation), relationship factors (duration of relationship, fear of partner’s reaction and HIV status of partner), barriers to HIV status disclosure ( fear of abandonment, fear of confidentiality, and fear of accusation of infidelity) and Outcomes of disclosure (acceptance, understanding, blame and violence). Information about disclosure status was documented by asking if the women had verbally revealed her HIV positive status to her sexual partner.

The questionnaire was translated into the Amharic (national language) by linguistic professionals. Matching was made on the exact fitness of the two versions. A pretest using the questionnaire was conducted among fifteen percent of the total sample size that was not included in the study.

The pretest as well as the study was done by trained data collectors (experienced nurse counselors who were working in the respective PMTCT centres) and any ambiguous and unsuitable questions were modified after the pretest had been conducted.

### Data analysis

Data was entered, cleaned and analyzed using SPSS version 15 computer software. Logistic regression model was used to examine the association between socio-demographic characteristics and relationship factors with HIV positive status disclosure to a partner and also the effect of disclosure on the intention to utilize PMTCT service. First factors related to the dependent variables were analyzed using bivariate analyses and those variables found significant at the bivariate were further entered and analyzed using logistic regression analysis.

### Ethical consideration

The study secured ethical clearance from the institutional review board of Addis Ababa University. All study participants were adequately informed about the purpose, method and anticipated benefit and risk of the study by the data collectors. Verbal consent was obtained from each participant and confidentiality of the study subjects was maintained.

## Results

### Socio demographic characteristics

A total of eight health centers were included in the study. The study population consisted of 112 HIV positive pregnant women, of whom 107 of them agreed to be interviewed, making the respondent rate of 95.5%. The remaining five women were not willing to participate in the study.

All 107(100%) participants were married and had one regular partner. Forty three (42.1%) respondents were in the age group of 25–29 years. Regarding the ethnicity of the respondents, the most numbering 32(29.9%) of the respondents were Amhara. Fifty two (48.6%) mothers had primary school education. Fifty nine (55.1%) of the respondents were house wives and unemployed. Forty-two (39.3%) of the respondents had a monthly income of less than 28 Dollars per month. The majority of women 71(66.4%) were followers of orthodox Christianity. Bivariate analysis showed that there were no statistically significant differences between those disclosing their status compared to those who had not with respect to these above-mentioned variables (see Table 1).

**Table 1:** Socio demographic characteristics of respondents by disclosure status (n = 107)

**Variables**	**Frequency**	**Percent (%)**
**Age:**		
15 – 19	6	5.6
20 – 24	26	24.3
25 – 29	43	42.1
30 – 34	23	21.5
35 – 39	35	5.7
**Religion:**		
Orthodox	71	66.4
Muslim	33	23.5
Protestant	3	2.8
**Educational level:**		
Illiterate (cannot read & write)	20	18.7
Can Read and write	9	8.4
Primary (1–8)	52	48.6
Secondary (9–12)	21	19.6
Diploma and above	5	4.7
**Marital status:**		
Married	107	100
Single, divorced/widowed	0	0
**Occupation:**		
Employed	16	15.0
House wife	59	55.1
Others	22	20.5
**Household income per month:**		
Less than 28 Dollars	42	58.3
28 – 56 Dollars	9	12.5
Above 56 Dollars	21	29.2

### Disclosure experience of HIV positive pregnant mothers

Seventy eight (73%) of the 107 respondents had disclosed their HIV sero-positive status to their sexual partner. Out of the 107 participants 42 (39.3%) of them had HIV positive partners. However, 25(23.4%) of them had HIV negative partners (discordant), 35(32.7%) had sex partners whose HIV status was not known, and only 5(4.7%) of the participants did not know their partner’s HIV status.

The reasons cited for disclosure were multiple and included concern for their partner’s health 52(66.7%), encouragement from their counselors 14(17.9%), and ethical responsibility 5(6.4%).

In addition, 24(51.1%), 8(17.0%), 6(12.8%), 2(4.3%) and 3(6.4%) of the respondents disclosed their HIV seropositive status to their mother, sisters, father, brothers & others respectively. Regarding time of disclosure the majority 51(65.4%) of the respondents disclosed their status within the first month after diagnosis (see Table 2).

**Table 2:** Sero-positive HIV status disclosure experience among HIV Positive pregnant women

**Variable**	**Number**	**%**
**Disclosure of HIV status to sexual partner (n = 107)**		
Yes	78	72.9
No	29	27.1
**Duration of time for disclosure since diagnose (n = 78)**		
< 1 month	51	65.4
1–2 months	10	12.8
3–4 months	11	14.1
>4 months	6	7.7
**Disclosure of HIV Status to others (n = 107)**		
Yes	43	40.2
No	64	59.8
**Disclosure of HIV status to family members (n = 43)**		
Father	6	12.8
Mother	24	51.1
Sister(s)	8	17.0
Brother(s)	2	4.3
Others	3	6.4
**Reasons for disclosure of HIV status to sexual partner (n = 78)**		
Ethical responsibility	5	6.4
Concern for partner’s health	52	66.7
Encouragement from counselors	14	17.9
Positive social support	1	1.3
**Discussion about VCT before HIV test (n = 78)**		
Yes	49	45.8
No	29	27.1

### Factors associated with disclosure of HIV status to sexual partner

Factors influencing disclosure of HIV positive status to sexual partner include duration of relationship with their partners, relationship before test, and prior discussion about VCT test. Among the 78 respondents who had disclosed, 52(48.6%) of them learnt their HIV positive status during ANC. Of eighty (74.8%) respondents who reported smooth relationship with their partners, 69(64.5%) had disclosed their HIV status while out of 20 (18.7%) who had disagreement with their partners, 9(8.4%) had disclosed their HIV status to their partner. Out of 107 study participants 42(39.3%) had HIV positive partners, and 25(23.4) HIV negative partners (discordant). Thirty-six (33.6%) out of forty-two study participants whose partner’s HIV status was known disclosed their HIV status to their partner and 22(20.6) out of twenty –five of the discordant disclosed their HIV status to their partner. Fifty one (47.7%) study participants had discussion about HIV testing prior to seeking VCT services but 56(52.3%) of the respondents did not. Those who had discussion with their partner to undertake the HIV test 49(45.8%) had disclosed their HIV positive status in comparison to those had no prior discussion about HIV test with their partners 29(27.1%). Eighty nine (83.2%) respondents lived with their partners for more than two years.

Strong association was found between prior communication about HIV testing with a partner and HIV-sero-status disclosure. Women who reported prior discussion about HIV testing were twelve times more likely to disclose their HIV status to partners than their counterparts (AOR 12.28, 95% CI 2.53-59.52).

Women who reported to have smooth relationship with the partner before the HIV test were six times more likely to disclose their HIV status to partners than those who reported living with their partners with disagreement (AOR 6.76, 95% CI 2.14-21.31) (Table 3).

**Table 3:** Factors influencing disclosure of HIV positive status to sexual partner among HIV Positive pregnant women

**Variables**	**Disclosed (N = 78)**	**Not disclosed (N = 22)**	***COR (95% CI)***	***AOR (95% CI)***
**Duration of relationship with partners:**				
0-2 yrs	12(15.4)	6(5.6)	0.69(5.05,103.0)	12.28 (2.53, 59.52)
>2 yrs	66(84.6)	23(21.5)	1.00	1.00
**Relationship before test:**				
Smooth relationship	69(64.5)	11(10.3)	7.6(4.51 – 34.87)	6.76 (2.14,21.31)
With disagreement	9(8.4)	11(10.3)	1.00	1.00
**Time of HIV Status learnt**				
During ANC	52(48.6)	22(20.6)	0.6(1.1-2.7)	0.2 (0.5-2.1)
Before ANC	26(24.3)	7(6.5)	1.00	1.00
**Discussion about VCT before HIV test:**				
Yes	49(64.5)	2(1.9)	22.8(5.0 – 103.0)	12.28 (2.53, 59.52)
No	29(8.4)	27(25.2)	1.00	1.00
**Partner’s HIV status**				
**Positive**	36 (33.6%)	22(20.6)	2.6 (0.62, 1.43)	0.79 (0.44, 1.45)
**Negative**	25(23.4)	40(37.4)		

COR Crude odds ratio, AOR Adjusted odds ration.

### Outcomes of HIV status disclosure

Positive outcomes reported by women in this study included increased support and less anxiety and increased intention to utilize PMTCT programs. Out of seventy-eight study participants, most 42(53.8%) reported that their partners reacted supportively to the disclosure of their HIV status, and only 15.4% reported any negative reaction from their partners. More than 44(56.4) study participants reported that they felt free following disclosure to their sexual partner. Although disclosure has a number of benefits, it is not without problems.

Negative outcomes from their partners included annoyed 2(2.6%), yelled at me 10(12.8%), worried about his HIV status 7(9.0%), blamed me to infect him 2(2.6%), talked about divorcing me 1(1.3%), don’t know 1(1.3) and no response 7(9.0%).

The feeling of respondents following disclosure was reported as worried 12(15.4%), felt free 44(56.4%), felt lighter 19(24.4%), and felt abandoned 3(3.8%) (Table 4).

**Table 4:** Outcomes of HIV status disclosure among HIV Positive pregnant women

**Variable**	**Number (%)**
**Partner’s reaction to HIV status disclosure**	
**(n = 78)**	42(53.0)
“Supportive”	7(9.0)
“Annoyed”	2(2.6)
“Yelled at me”	10(12.8)
“Worried about his own HIV status”	7(9.0)
“Blamed me to infect him”	2(2.6)
“Talked about divorcing me”	1(1.3)
“Don‘t know”	7(9.0)
No response	
**Feeling of respondents after disclosure of HIV positive status: (n = 78)**	
“Worried”	12(15.4)
“Felt free”	44(56.4)
“Felt lighter”	19(24.4)
“Felt abandoned”	3(3.8)

### The effects of HIV status disclosure on intension to utilize PMTCT service

Measures cited by respondents to prevent mother to child transmission of HIV includes starting ART 43(40.2%), and bringing new born for ART prophylaxis 71(66.4) (AOR1.45 95% CI 0.44-5.12, 4.74 95% CI 1.24 – 18.14, respectively) (Table 5).

**Table 5:** Effects of HIV status disclosure on intension to utilize PMTCT service

**Variable**	**Disclosed (n = 78)**	**Didn’t disclose (n = 29)**	**COR (95% CI)**	**AOR (95% CI)**
Start ART after disclosure of the test?				
Yes	43(40.2)	9(8.4)	2.7(1.10-6.75)	1.45(0.44-5.12)
No	35(32.7)	20(18.7)	1.00	1.00
**Timing of new-born visit for ART prophylaxis:**				
Within one day after delivery	68(63.5)	15(14.0)	6.3(3.1-12.7)	1.9(0.6-4.9)
Two days or more after delivery	10(9.3)	14(13.1)	1.00	1.00
Do you bring the new born for ART prophylaxis if you deliver at home?				
Yes	71(66.4)	16(15.0)	8.24(2.84 – 23.95)	4.74(1.24 – 18.14)
No	3(2.8)	6(5.6)	1.00	1.00
Do not know	4(3.7)	7(6.5)		
After knowing your HIV serostatus, have you started practicing safer sex?				
Yes	54[50.4]	4[3.7]	14.06(0.42, 0.92)	0.71(0.40, 1.27)
No	24[22.1]	25[23.4]	1.00	1.00

COR Crude odds ratio, AOR Adjusted odds ratio.

## Discussion

This study aimed to determine prevalence of, barriers to and independent predictors of disclosure of HIV positive status to a sexual partner among the pregnant women attending ANC in selected Health Centers in Addis Ababa, Ethiopia.

Seventy eight (73%) of the 107 respondents had disclosed their HIV sero-positive status to their sexual partner. This is higher than a study from Tanzania (16.7%), Burkina Faso (31.6%) and Kenya (65%) [13-15]. The higher rate of disclosure in this study may be related to advances in PMTCT and antiretroviral treatment programs. These advances have led to decreased HIV associated stigma in the community, recognition among who test positive of the benefits of disclosure and increased expertise of counselors who work with these clients. However, 27.1% of the people interviewed in this study didn’t disclose their status to their partner. Counseling of HIV positive pregnant women who did not disclose their HIV status during ANC would help them to disclose their status. Service providers at PMTCT clinics should ensure that all efforts are made to counsel HIV positive pregnant women about disclosure of their status to their sexual partner from the beginning in every PMTCT interventions.

Our estimate of delayed disclosure is somehow lower than those reported in another studies. In a PMTCT study conducted in Tanzania, only 22% of women disclosed to someone within 18 months period following diagnosis. Other study conducted in Burkina Faso [14] among 79 HIV Positive pregnant women at ANC 31.6% had shared their status with their partner at all average duration of 8 months after VCT. But in relation to time of disclosure, in this study about two-third of the women (65.4%) who ever disclosed to a partner their HIV positive status did so with in less than one month of knowing their HIV status. However, 12.8% of the disclosures were delayed by 1-2 months, 14.1% by 3–4 months, and 7.7% by more than four months respectively. These study participants might have at least one sexual contact with their partner before disclosure. This raises the possibility of transmission risk if condoms were not used and may limit the beneficial aspect of disclosure making negotiating safer sex difficult and perhaps putting the partner at risk of infection.

Inconsistent with other studies [13-15] the proportion of disclosure to partners (73%) was found to be far greater than disclosure to family members (40.2%). One possible explanation for the low disclosure rate to family members is the difference in the concern about the health condition of one’s partner. In this study, it was found that concern for partner’s health was the major reason cited for disclosing to sexual partners. This result is also similar to other studies [13].

Despite the high rate of HIV status disclosure, a significant proportion (14.0%) had sex partners whose HIV status was not known and some (4.7%) of the participants did not know their partner’s HIV status. The silence of the partners could be either acknowledging that she is already infected with the HIV or the result of the emotional rejection of the partner. These findings remind us that individuals with unknown or negative HIV status should be educated about the need for consistent use of condoms with their sexual partners.

In this study, prior discussion with one’s partner to undertake HIV test and having smooth relationship with one’s partner is significantly associated with disclosure. This might help the study participants to anticipate their partner’s reaction and is quite important in women’s negotiation and decision making around disclosure.

This finding agrees with many other studies that showed people who reported to have good relationship and discussion prior to HIV testing are more likely to disclose their HIV status [16-18].

The majority of women in this study reported that their partners reacted supportively to disclosing of their HIV status. Negative out comes reported by women included annoyed at me, yelled at me, worried about his HIV status, blamed me to infect him & talked about divorcing me. The reasons cited for non-disclosure were fear of divorce & violence, fear of abandonment, fear of confidentiality, and fear of accusation of infidelity. This finding is in agreement with other studies [18]. Women who experienced fear of negative consequences of disclosure were less likely to disclose their HIV positive status [19]. This could be explained that attitudinal outcome beliefs such as these are extremely important in predicting behavior [20].

In this study, women who disclosed their status to their partner were found to be more likely to participate in Prevention of Mother to Child Transmission (PMTCT) programs. HIV positive pregnant women that reported disclosure to their partner in this study were found to bring their new born for ART prophylaxis if delivered at home four times compared to those who did not disclose. We found a very strong statistical association between having disclosed one’s HIV status to partner and engaging in safe sex practice. Participants who had disclosed to sex partners were 14 times more likely to practice safe sex than those participants who did not disclose their HIV sero-status. This could be explained that disclosure facilitates other behaviors that may improve utilization of PMTCT programs and HIV/AIDS prevention. In the case of PMTCT, women who have knowledge about modes of MTCT of HIV infection may be more likely to participate in prevention of mother- to- child Transmission (PMTCT) programs.

In addition, women who disclose their status are in a better position in terms of reproduction choices as well as psychosocial support. This study showed that women who disclosed their results need to take ART medication themselves and give it to their new-born, breast feed exclusively for 6 months and deliver in the health institutions to reduce the risk of mother to child transmission of HIV Infection. This is in line with other studies [7]. Pregnant women who disclosed to their partners reported more frequent condom use or abstinence. After disclosure, partners often seek voluntary counseling and testing if the partner tests negative he can protect himself by using condoms during sex. Thus, risks of exposure to HIV through unprotected sex are far less common for participants who had disclosed their status to their main partners. These findings are similar with many other studies [16,21,22].

Disclosure was also associated with less anxiety and increased social support [23,24]. Similarly, more than half of the women in this study (56.4%) reported that they felt free following disclosing of their HIV status to their main partners.

The findings of this study should be interpreted in the light of its limitations. The result of this study depended on self-report of a sensitive topic disclosure of HIV status. As a result there may be influence of social desirability. However, measures were taken to reduce this bias by granting confidentiality, maintaining privacy and explaining the purpose of the study to participants. In addition, the high response rate is one of the strength of this study. The findings of this study may mainly be applied to similar population in urban/periurban areas in Ethiopia.

## Conclusions

This study revealed that HIV positive status disclosure among pregnant women is high. Although most participants disclosed their HIV sero-positive status, lack of disclosure by some women resulted in a limited ability to participate in PMTCT programs. Thus, assertiveness and improved communication skills training should be offered to HIV positive pregnant mothers and be reinforced during on-going counseling.

## Competing interests

The authors declare that they have no competing interests.

## Authors’ contributions

EG and AC participated from the inception of the research idea to proposal development, data collection, analysis and preparation & revision of the manuscript and TA participated in the preparation of the manuscript for publication. All authors read and approved the final version of the manuscript.

## Pre-publication history

The pre-publication history for this paper can be accessed here:

http://www.biomedcentral.com/1471-2458/13/765/prepub

## References

[B1] WHO Gender dimensions of HIV status disclosure to sexual partners: rates, barriers and outcomes A review paper 2004

[B2] Ministry of Health Ethiopia & Federal HIV/AIDS Prevention and Control Office Single point HIV prevalence estimate 2007 Addis Ababa, Ethiopia

[B3] Central Statistical Agency and ORC Macro Ethiopia demographic and health survey 2005 2006 Addis Ababa, Ethiopia and Calverton, Maryland, USA

[B4] Deribe et al Disclosure experience and associated factors among HIV positive men and women clinical service users in southwest Ethiopia BMC Publ Health 2008 8 81 doi:10.1186/1471-2458-8-81 10.1186/1471-2458-8-81 PMC2275263 18312653

[B5] United Nations Joint Program on AIDS: *http://www.Unaids.org International center for AIDS care and treatment programs(ICAP).* Columbia University’s mailman School of Public Health

[B6] Joint United Nations Programme on HIV/AIDS (UNAIDS) World health organization (WHO) AIDS epidemic update: December 2006 Geneva: UNAIDS/WHO

[B7] Medley A Garcia-Moreno C McGill S Maman S Rates, barriers and outcomes of HIV serostatus disclosure among women in developing countries: implications for prevention of mother-to-child transmission programmes Bull World Health Organ 2004 82 299 307 15259260 PMC2585956

[B8] Ethiopian AIDS resource centerhttp://www.etharc.org. accessed on December 2010

[B9] Federal Ministry of health National HIV/AIDS prevention and control office AIDS in Ethiopia. Sixth report

[B10] Bennets A Determinants of depression and HIV related worry among HIV positive women who have recently given birth, Bangkok, Thailand Soc Sci Med 1999 49 737 749 10.1016/S0277-9536(99)00108-2 10459886

[B11] Naver L Perinatal HIV-1 infection, aspects on clinical presentation, viral dynamics and epidemiology Thesis 2004 Stockholm: Karolinska University press

[B12] United Nations AIDS AIDS epidemic up dates 2005 Geneva: UNAIDS

[B13] Kilewo C Massawe A Lyamuya E HIV counselling and testing of pregnant women in sub-Saharan Africa: experiences from a study on the prevention of mother-to-child HIV-1 transmission in Dar es salaam, Tanzania J AIDS 2001 28 458 462 10.1097/00042560-200112150-00009 11744835

[B14] Issiaka S Catoux M Ky-Zerbo O Living with HIV: women’s experience in Burkina Faso, West Africa AIDS Care 2001 13 123 128 10.1080/09540120020018224 11177469

[B15] Farquhar C Ngacha D Bosire R Prevalence and correlates of partner notification regardingHIV-1 in an antenatal setting in Nairobi Kenya. Durban: XIII International AIDS Conference 2000 9–1 4 381

[B16] Kassaye KD Associated factors and out Comes of disclosing HIV sero-positivity status to sexual partners among women in mettu and gore towns, illubabor zone southwest Ethiopia Ethiop J Health Dev 2005 19 126 13

[B17] Kilmarx P Hamers F Experience and perspectives of HIV infected and sexually transmitted disease clinic patients after post test counselling Sex Transm Dis 1998 25 28 37 10.1097/00007435-199801000-00007 9437782

[B18] Simoni J HIV disclosure among women of African decent: association with coping, social support, and psychological adaptation AIDS Behav 2000 4 147 158 10.1023/A:1009508406855

[B19] Lester P The consequences of a positive prenatal HIV antibody test for women J Acquir Immune Defic Syndr Hum Retroviral 1995 10 341 349 7552496

[B20] Lie GT Biswalo PM HIV positive patient’s choice of a significant other to be informed about the HIV test result: finding from an HIV/AIDS counselling program in the regional hospital of arusha and Kilimanjaro, Tanzania AIDS care 1996 8 285 296 10.1080/09540129650125713 8827121

[B21] Antelmen G Smith Fawzi MC Predictors of HIV −1 serostatus disclosure: a prospective study among HIV- infected pregnant women in Dar es salaam, Tanazania AIDS 2001 15 1865 1874 10.1097/00002030-200109280-00017 11579250 PMC6261328

[B22] Mackneil JM Is care and support associated with preventive behaviour among people with HIV? AIDS Care 1999 11 537 546 10.1080/09540129947695 10755029

[B23] Vander Straten A Couple communication, sexual coercion and HIV risk reduction in Kigali, Rwanda AIDS 1995 9 935 944 10.1097/00002030-199508000-00016 7576330

[B24] Temmarman M The right not to know HIV test results The Lancet 2005 345 969 970 10.1016/s0140-6736(95)90707-6 7619122

